# Detection of novel fusion-transcripts by RNA-Seq in T-cell lymphoblastic lymphoma

**DOI:** 10.1038/s41598-019-41675-3

**Published:** 2019-03-26

**Authors:** Pilar López-Nieva, Pablo Fernández-Navarro, Osvaldo Graña-Castro, Eduardo Andrés-León, Javier Santos, María Villa-Morales, María Ángeles Cobos-Fernández, Laura González-Sánchez, Marcos Malumbres, María Salazar-Roa, José Fernández-Piqueras

**Affiliations:** 1 Department of Cellular Biology and Immunology, Severo Ochoa Molecular Biology Center (CBMSO), CSIC-Madrid Autonomous University, Madrid, 28049 Spain; 2 Institute of Health Research Jiménez Diaz Foundation, Madrid, 28040 Spain; 3 Consortium for Biomedical Research in Rare Diseases (CIBERER), Spain. Carlos III Institute of Health, Madrid, 28029 Spain; 4 Cancer and Environmental Epidemiology Unit, National Center for Epidemiology, Carlos III Institute of Health, Madrid, 28029 Spain; 5 Consortium for Biomedical Research in Epidemiology and Public Health (CIBERESP), 28029, Madrid, Spain; 6 Bioinformatics Unit, Structural Biology and Biocomputing Programme, Spanish National Cancer Research Center (CNIO), Madrid, 28029 Spain; 7 Bioinformatics Unit, Instituto de Parasitología y Biomedicina “López-Neyra”, Consejo Superior de Investigaciones Científicas (IPBLN-CSIC), PTS Granada, Granada, 18016 Spain; 8 Cell Division and Cancer Group, Molecular Oncology Programme, Spanish National Cancer Research Centre (CNIO), Madrid, 28029 Spain

## Abstract

Fusions transcripts have been proven to be strong drivers for neoplasia-associated mutations, although their incidence in T-cell lymphoblastic lymphoma needs to be determined yet. Using RNA-Seq we have selected 55 fusion transcripts identified by at least two of three detection methods in the same tumour. We confirmed the existence of 24 predicted novel fusions that had not been described in cancer or normal tissues yet, indicating the accuracy of the prediction. Of note, one of them involves the proto oncogene *TAL1*. Other confirmed fusions could explain the overexpression of driver genes such as *COMMD3-BMI1*, *LMO1* or *JAK3*. Five fusions found exclusively in tumour samples could be considered pathogenic (*NFYG-TAL1*, *RIC3-TCRBC2*, *SLC35A3-HIAT1*, *PICALM MLLT10 and MLLT10-PICALM*). However, other fusions detected simultaneously in normal and tumour samples (*JAK3-INSL3*, *KANSL1-ARL17A/B and TFG-ADGRG7*) could be germ-line fusions genes involved in tumour-maintaining tasks. Notably, some fusions were confirmed in more tumour samples than predicted, indicating that the detection methods underestimated the real number of existing fusions. Our results highlight the potential of RNA-Seq to identify new cryptic fusions, which could be drivers or tumour-maintaining passenger genes. Such novel findings shed light on the searching for new T-LBL biomarkers in these haematological disorders.

## Introduction

Precursor T-cell lymphoblastic neoplasms are aggressive haematological malignancies that most often manifest with extensive marrow and blood affectation (T-cell acute lymphoblastic leukaemia or T-ALL) or less commonly as a thymic mass with limited bone marrow infiltration (<25%) (T-cell lymphoblastic lymphoma or T-LBL). Immunophenotypic analyses revealed that T-ALL tend to show a more immature profile and gene expression profiling studies segregated these subtypes into two clusters, suggesting underlying biological differences^1^. T-LBL subtype represents the second most frequent type of non-Hodgkin’s lymphoma (NHL) in children and adolescents^2^.

Fusions transcripts result from the exchange of coding or regulatory DNA sequences between different genes. They are usually strong driver neoplasia-associated mutations, sometimes even pathognomonic, with great importance in the development of tumours and in the medical practice^3^. Recent advances in deep-sequencing technologies (in particular Whole-Genome DNA sequencing, and Whole Transcriptome Sequencing (RNA-Seq)) have greatly facilitated uncovering novel fusion transcripts in cancer cells, showing that they are much more common than previously presumed. Therefore, even if many of these fusions are probably little or no pathogenic, the search for new fusions should be a priority task^3,4^.

Gene fusions usually are hallmarks of cancer and this is especially notorious in haematological malignancies^5^. Of note, T-ALL is caused by mutations affecting multiple oncogenes, tumour-suppressor genes, and genes involved in chromatin remodelling, but also by large chromosomal deletions, amplifications and chromosomal translocations, which involve the T-cell receptor (TCR) loci or generate in-frame fusion genes encoding chimeric proteins with oncogenic properties^6^. Two comprehensive analyses of fusion-transcripts have been reported in T-cell acute lymphoblastic leukaemia^7,8^.

Since RNA-Seq is a very promising technology for the identification of cryptic structural variations, and chromosomal instability is a hallmark in cancer development, the aim of this work was to gain insights into the transcriptome landscape of T-LBL, a subtype of T-cell lymphoblastic neoplasms that has attracted much less attention from the researchers in this field. We examined RNA Sequencing data using three different pipeline/fusion-mining tools to explore novel cryptic structural variations characteristic of this neoplasia.

## Results

### There exist noticeable differences between predictive algorithms in identifying fusion transcripts

**Figure 1:**
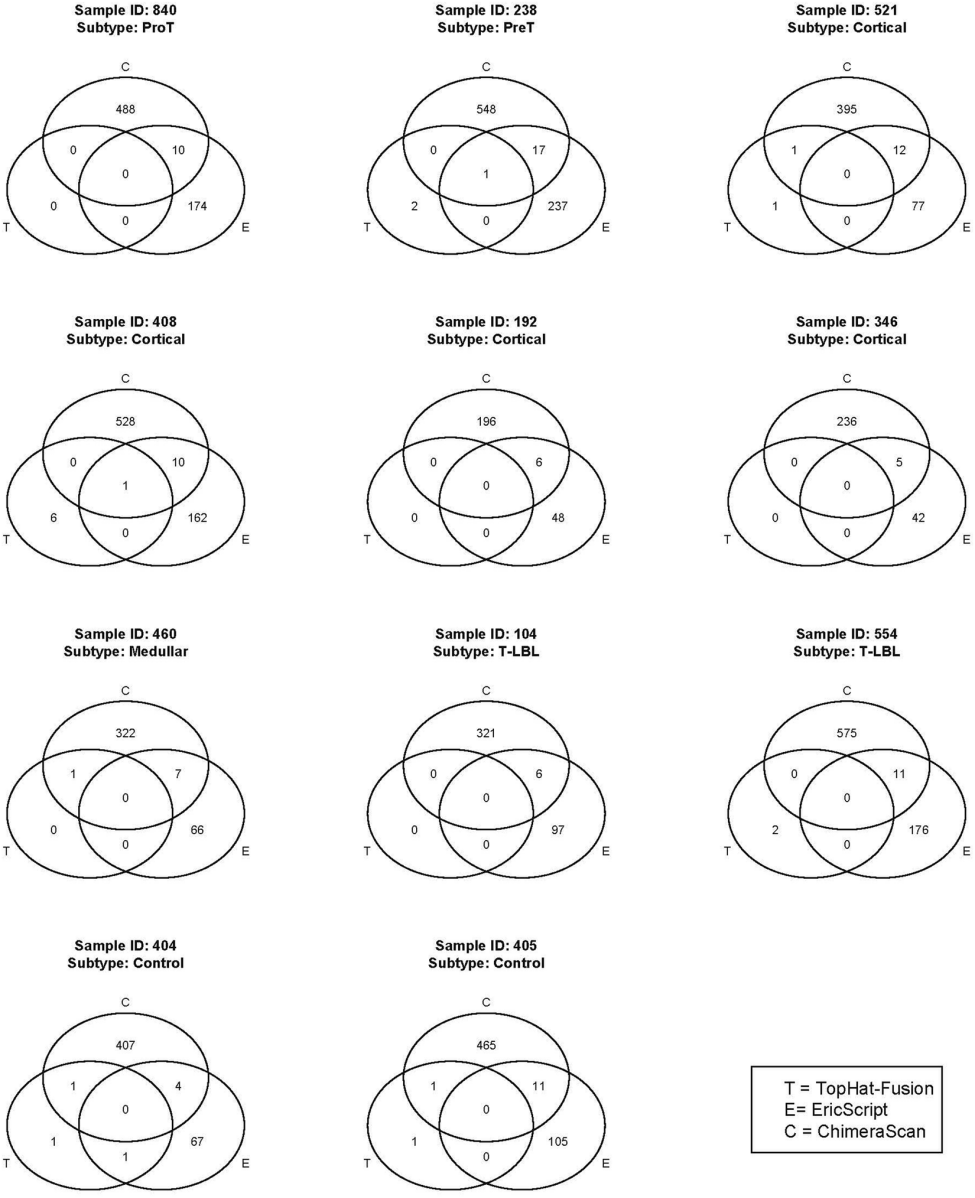
Venn diagrams showing the number of fusion transcripts identified by the three detection methods (*TopHat-fusion*, *Chimerascan and EricScript*) in nine T-LBL samples and two control thymuses.

For this study, we initially analyzed nine human T-LBL samples (discovery cohort) (Supplementary Table S1) and two control non-pathological thymuses of fetal origin by massive sequencing of mRNAs (RNA-Seq). Selection of fusion transcripts was based on optimal RNA quality and significant scores of three detection tools/algorithms. Total fusion transcripts identified by each of the three methods are depicted in Supplementary Table S2. As expected, *TopHat-Fusion* identified the lower number of fusion transcripts oscillating between zero (in tumours 104, 192, 346, 840) and seven fusions (in tumour 408). The two other tools identified a considerably higher number of fusion transcripts, which oscillated, in the case of *ChimeraScan*, between 202 in tumour 192 up to 586 fusions in tumour 554, and in the case of *EricScript* between 47 fusions in tumour 346 up to 255 fusions in tumour 238. Of note, only two fusions, *ZMYM2-FGFR1* (in tumour 408) and *TFG-ADGRG7* (in tumour 238), were identified in the same tumour by the three detection methods. Interestingly, two control thymuses also exhibited a high number of fusions, some of them (*KANSL1-ARL17A/B*) also identified in tumours (Fig. 1).

### Selection of high-confident fusion transcripts

To select high-confident fusion transcripts, therefore avoiding false positives, we considered only predictions supported by at least two detection tools in the same sample. After applying these stringent criteria, we selected 55 fusion transcripts across all tumour samples (Tables 1 and S3). Accordingly to the Atlas of Genetics and Cytogenetics in Oncology and Haematology^7,9–12^, 24 fusions had not been described in cancer or normal tissues yet, 17 had been identified only in non-neoplastic or normal tissues, 1 had been shown exclusively in T-cell lymphoblastic leukaemia/lymphoma (*RIC3-TRBC2*), 1 only in T-cell lymphoblastic neoplasms, 1 exclusively in cancers other than T-cell lymphoblastic neoplasms (*MRPS16-TTC18*), 10 in cancer others than T-cell lymphoblastic neoplasms and in non-neoplastic or normal tissues and 2 in T-cell lymphoblastic neoplasms and other cancers (*PICALM-MLLT10* and *MLLT10-PICALM*).

**Table 1:** Selected fusion transcripts found in nine T-LBL samples, classified according to their immunological data.

Fusion	ProT	PreT	Cortical				Medullar	T-LBL		Control		Previous reports*
	840	238	521	408	192	346	460	104	554	404	405	
CLN6;CALML4^✢,a^		●	●●				●●	●●			●●	0
GXYLT2;PPP4R2^✢,a^		●●	●●	●				●●	●●			0
XPO7;NPM2^✢,a^								●●				0
DNAJC4;VEGFB^✢,a^					●●							0
UTP6;COPRS^✢,a^					●●							0
TUT1;EEF1G^✢,a^		●●	●●	●●	●●						●●	0
OPN3;CHML^✢,a^		●●	●	●					●	●	●	0
KANSL1;LRRC37A^✢,b^		●●		●●			●		●			0
SAV1;GYPE^✢,c^	●●	●●							●●		●	0
GALT;IL11RA^✢,a^		●●	●●	●●								0
DNAAF3;TNNI3^✢,a^	●●	●●										0
SSSCA1;FAM89B^✢,a^		●		●		●●	●		●	●●	●●	0
KANSL1;ARL17B^✢,a^			●			●				●●	●	0
GPC2;GAL3ST4^✢,a^				●						●	●●	0
DPP6;ACTR3B^✢,a^											●●	0
GAL3ST4;C7orf43^✢,a^				●●	●							0
SNX29;PLA2G10^✢,b^							●●					0
BPTF;LRRC37A2^✢,b^		●					●●				●	0
SPN;QPRT^✢,a^	●	●	●●	●				●	●			0
NFYC;TAL1^✢,b^			●●									0
PTCRA;CNPY3^✢,a^									●●	●	●	0
PPRC1;NOLC1^✢,a^				●					●●			0
KANSL1;LRRC37A2^✢,b^		●		●					●●			0
DTX2;UPK3B^✢,a^	●●	●		●	●		●		●	●	●	0
HAUS4;PRMT5^a^	●							●●				1
FAM175A;HELQ^b^			●					●●				1
RRM2;C2orf48^a^	●●	●●	●●	●●	●●	●●	●●	●	●	●●	●●	1
VAMP8;VAMP5^c^	●●●	●●			●●		●●			●●		1
ADSL;SGSM3^a^	●	●●		●	●		●			●		1
NSUN4;FAAH^a^	●●	●●	●									1
VPS45;PLEKHO1^a^		●		●	●	●●	●●				●	1
SLC35A3;HIAT1^a^			●						●●		●●	1
SMG5;PAQR6^a^				●●								1
RNASET2;RPS6KA2^a^	●			●●				●				1
CNPY2;CS^a^		●	●●	●	●	●	●	●	●			1
FAM117A;SLC35B1^a^	●		●●								●	1
DRAXIN;AGTRAP^a^									●●			1
GLYCTK;DNAH1^a^							●		●●			1
PRKAA1;TTC33^a^		●		●				●	●●			1
UBA2;WTIP^a^	●●		●					●				1
DUS3L;PRR22^a^	●●								●			1
RIC3;TRBC2^✢,c^							●●					2
MRPS16;TTC18^a^											●●	4
DHRS1;RABGGTA^a^	●●	●●	●●	●●	●●	●	●●	●●	●●	●●	●●	5
TFG;ADGRG7^✢,a^		●●●										5
C15orf57;CBX3^c^		●●		●					●			5
ADCK4;NUMBL^a^	●	●●		●●						●	●	5
JAK3;INSL3^✢,a^	●	●●	●			●●						5
CTBS;GNG5^a^		●		●		●●	●				●●	5
KANSL1;ARL17A^✢,a^			●●			●				●●	●●	5
ZMYM2;FGFR1^✢,c^				●●●								5
PRIM1;NACA^a^	●		●●	●	●	●	●	●	●	●	●	5
SIDT2;TAGLN^a^	●●	●										5
PICALM;MLLT10^c^		●●										6
MLLT10;PICALM^c^		●●										6

Samples 404 and 405 are two normal thymuses. Fusion transcripts identified by one, two or the three detection methods are indicated with one, two or three dots, respectively. Previous reports: 0, not found in the Atlas of Genetics and Cytogenetics in Oncology and Haematology; 1, only found in non-neoplastic or normal tissues; 2, only found in T-cell lymphoblast leukaemia/lymphoma; 3, found in T-cell lymphoblastic leukaemia/lymphoma and in non-neoplastic or normal tissues; 4, only found in other cancers; 5, found in other cancers and in non-neoplastic or normal tissues; 6, found in T-cell lymphoblastic leukaemia/lymphoma and other cancers. Superscript letters indicated the type of fusion (a, read-though; b, intra-chromosomal; c, inter-chromosomal). ^✢^indicated fusion transcripts confirmed by Sanger sequencing.

**Figure 2:**
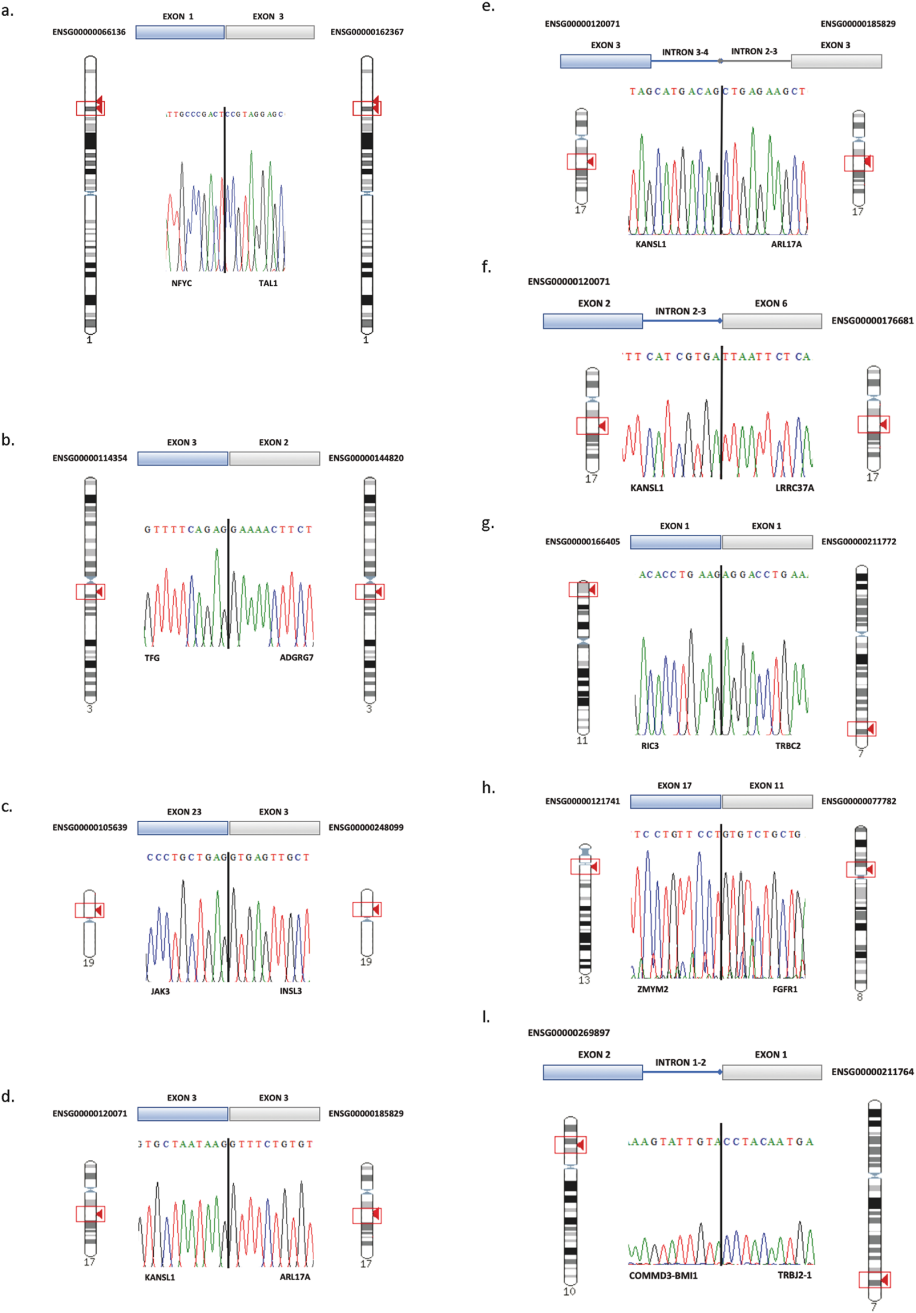
Validation of the fusion junction sequences of selected fusion transcripts by Sanger sequencing. Vertical black-bars indicate the fusion junctions. All validations were performed at the transcript level, with the exception of *KANSL1-ARL17A* that was validated both at transcript and genomic levels (d and e, respectively).

Supportively, RT-PCR and Sanger sequencing using the primers listed in Supplementary Table S4 confirmed the 24 novel fusions, at the transcriptional level in lymphoma samples of the discovery cohort and other tumours in an extended cohort (Supplementary Tables S1 and S5). Of note, 10 out of the 24 novel fusions were confirmed in all control and tumour samples (Supplementary Fig. S1a) and its incidence on tumour development should be negligible; 13 out of 24 were only confirmed in a fraction of tumour and control samples and could be considered polymorphisms, in any case with limited functional capacity (Supplementary Fig. S1b); and only the *NFYC-TAL1* fusion was confirmed exclusivelly in tumour 521 and could be considered as pathogenic (Fig. 2a). Notably, the *NFYC-TAL1* fusion is an intra-chromosomal fusion transcript that juxtaposes the exon 1 of the nuclear transcription factor Y, gamma (*NFYC)* to the exon 3 of the T-cell acute lymphocytic leukemia 1 (*TAL1*), producing an in-frame chimeric transcript that uses a new ATG site but rertains the bHLH domain. Interestingly, tumour 521 exhibits a significant increase in the levels of *TAL1* expression (FPKM, Fragments Per Kilobase of exon per Million reads vary from 0.47 in controls to 6.06 in the tumour,; log_2_FoldChange = 3.6) and in the levels of *TP53* expression (FPKM values varying from 45.5 in controls to 79.8 in the tumour sample).

In the remaining fusion transcripts other fusions could explain the overexpression of driver genes such as *COMMD3-BMI1*, *LMO1* or *JAK3*. Four fusions found exclusively in tumour samples could be considered pathogenic (*RIC3-TCRBC2*, *SLC35A3-HIAT1*, *PICALM MLLT10 and MLLT10-PICALM*). However, other fusions detected simultaneously in normal and tumour samples (*JAK3-INSL3*, *KANSL1-ARL17A/B and TFG-ADGRG7*) could be cancer predisposition ones probably involved in tumour-maintaining tasks.

Seven fusion transcripts deserved to be highlighted as representatives of different types and consequences (Fig. 2b–i). The *TFG-ADGRG7* fusion was identified exclusively in tumour 238 (a preT-immature T-LBL detected in an adult female patient) by the three different detection tools. It is a fusion transcript predicted to result from an inter-genic splicing (read-through) that produces an in-frame chimeric transcript/protein after joining the exon 3 of the TRK-fused gene (*TFG*), a known translocation target, to the exon 2 of the Adhesion G Protein-Coupled Receptor G7 (*ADGRG7*, alias *GRIP128*), involved in G-protein coupled receptor activity and trans-membrane signalling receptor activity, both genes located on the forward strand of DNA in chromosome 3 (Fig. 2b). Interestingly, the fusion transcript is expressed at higher levels (FPKM value: 46.67) than the transcripts corresponding to the two involved genes (TFG: 35.21; ADGRG7: 16.51) (Supplementary Table S6).

The *JAK3-INSL3* fusion is a read-trough transcript fusion we identified by RNA-Seq in tumours 238 (preT-immature T-LBL in an adult female), 346 (common/cortical T-LBL in adult male), 840 (proT-immature T-LBL in a paediatric male). However, RT-PCR-Sanger allows us to detect this fusion in other samples (408, 521, and 554). This fusion is the product of a read-through (inter-genic/*cis*-splicing) event between two adjacent genes on the reverse strand of DNA of chromosome 19, which juxtaposes exon 23 (the last coding exon) of Janus Kinase 3 (*JAK3*) with the third exon of Insulin Like 3 (*INSL3*), resulting in a chimeric transcript that replaces the 3′UTR of *JAK3* with that of *INSL3* (Fig. 2c). Notably, RNA-Seq expression in tumour 238 revealed an increase in the relative-levels of expression of the fusion transcript (FPKM value: 133.77) with respect to *JAK3* (FPKM value: 105.96) (Supplementary Table S6). Furthermore the levels of expression of total *JAK3* increases significantly in tumours 554 (FPKM values from 25,19 in controls to 53.80; log2FC = 1.09) and 840 (FPKM values from 22.60 in controls to 68.30; log2FC = 1.5).

The fusion between two other adjacent genes KAT8 regulatory NSL complex subunit 1 (*KANSL1*) and ADP-ribosylation factor-like 17 A/B (*ARL17A*), both at the reverse strand of chromosome 17, was identified in tumour 521 (a cortical T-LBL in a paediatric male patient) and in the two normal thymuses (404 and 405). A variant involving the paralog *ARL17B* gene was also identified in a normal thymus (404). As the easiest possibility, we might think that these fusions could result from a read-through that juxtaposes the exon 3 of *KANSL1* to the exon 3 of the partner gene *ARL17A/B*. However, this assumption is certainly improbable. This is firstly because it would require an inversion of the genomic structure to place the *ARL17A* gene downstream *KANSL1*, and additionally by the existence of multiple *KANSL1-ARIL1A* isoforms^13^. Therefore, it is reasonable to think that this fusion results from two steps: first an inversion and subsequently a fusion event between a 5′breakpoint downstream of the *KANSL1* exon 3 and a 3′ breakpoint occurring upstream of the *ARL17A* exon 3 (Fig. 2d). Sanger sequencing of cloned/genomic PCR fragments confirmed the existence of this gene fusion at the genomic level (Fig. 2e). Another intra-chromosomal fusion connects *KANSL1* on the reverse strand of chromosome 17, and *LRRC37A* (leucine rich repeat containing 37 member A) a new adjacent partner on the forward strands of the same chromosome. This fusion was identified in tumours 238 (preT-immature T-LBL in an adult female), and 408 (cortical/common T-LBL in an adult female*)*. A variant involving the paralog *LRRC37A2* was found in tumour 554 (Fig. 2f).

*ZYMYM2-FGFR1* fusion was identified exclusively in tumour 408, a cortical/common T-LBL detected in an adult female patient. This is an inter-chromosomal fusion that involves the zinc finger MYM-type containing 2 (*ZMYM2*) gene at chromosome 13 and the Fibroblast Growth Factor Receptor-1 (*FGFR1*) gene at chromosome 8, resulting in a chimeric protein that joint the proline-rich region of ZMYM2- responsible for protein oligomerization- and the tyrosine kinase domain of FGFR1 (Fig. 2g).

**Figure 3:**
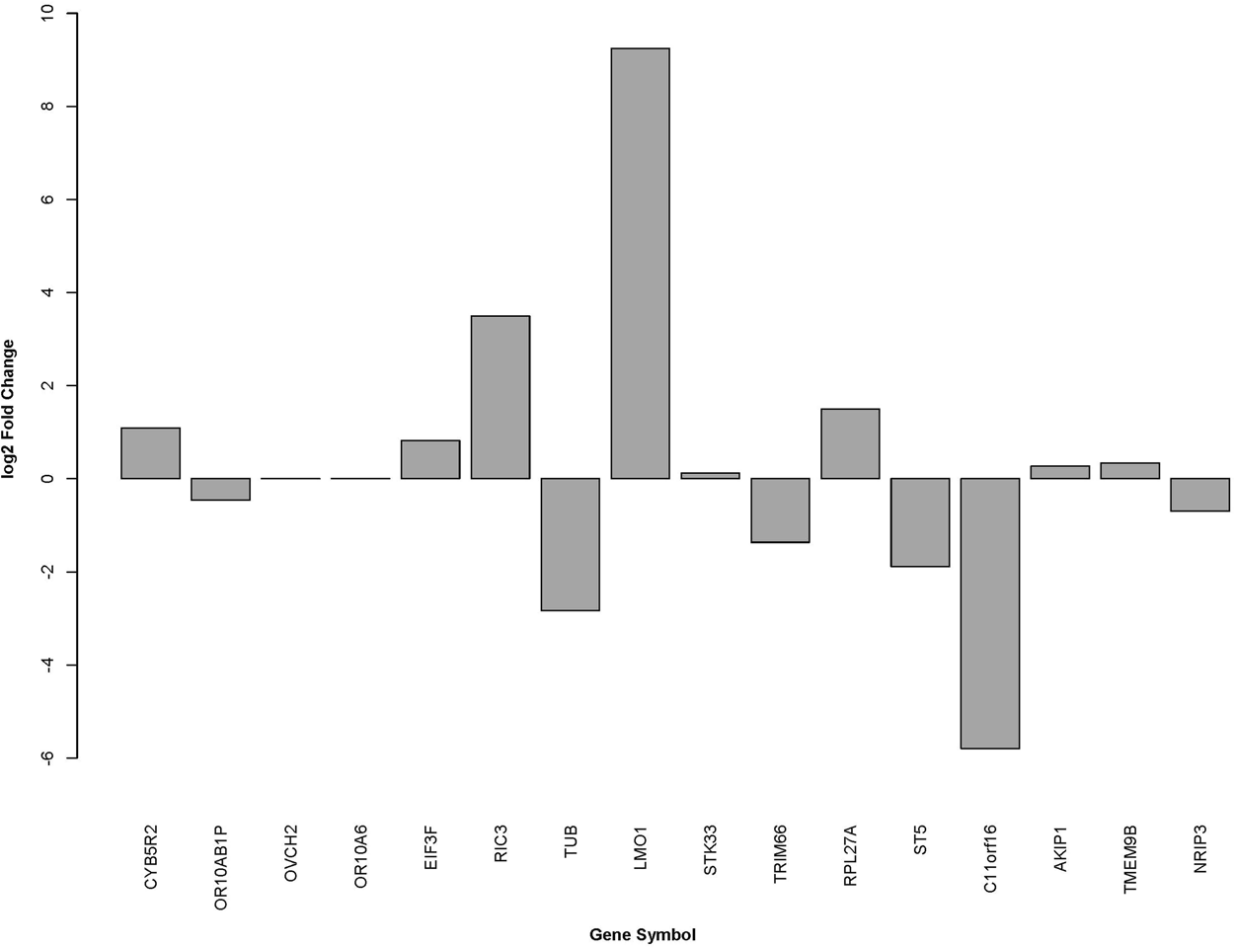
Levels of expression of 16 genes around the breakpoint of the *RIC3-TCRBC2* fusion. *LMO1* gene showed significant over-expression in comparison with its level of expression in control thymuses.

Among the transcript fusions that do not encode chimeric proteins (out-of-frame) we identified a paediatric mature T-LBL in a male patient (460) exhibiting the fusion of Resistant To Inhibitor Of Cholinesterase 3 (*RIC3*) gene at chromosome 11 to T-cell Receptor Beta Constant 2 (*TCRBC2*) gene located at chromosome 7 (*RIC3-TCRB2*) (Fig. 2h). Interestingly, *RIC3* encodes a member of a protein family that has never been associated to cancer. Therefore, the candidate gene in this fusion might be another gene located upstream or downstream of the breakpoint, whose regulation was affected by the regulatory sequences of *TCRBC2*. For this purpose we searched for the levels of expression of 16 genes around the breakpoint of the fusion. Of note, only the LIM domain only 1 (*LMO1*) gene showed significant over-expression with a fold change (log2 fold change) greater than 9.24 in comparison with its level of expression in control thymuses (Fig. 3).

### COMMD3-BMI1-TRBJ2-1 could be another relevant fusion

Since *TopHat-Fusion* seems to be very restrictive and quite reliable on detecting fusion transcripts^14^ we also validated the complex fusion *COMMD3-BMI1-TRBJ2-1* identified exclusively by this tool in tumour 554. This fusion juxtaposes TCR regulatory elements to the intron 1-2 of *COMMD3-BMI1* gene that, in turn, resulted from a read-through transcription between the COMM domain-containing protein 3 (*COMMD3*) and B lymphoma Mo-MLV insertion region 1 homolog (*BMI1*) genes. Notably we demonstrate the existence of this complex fusion, not only in tumour 554 but also in a second tumour (840) where it had not been predicted (Fig. 2i). As expected the levels of expression of *BMI1* increases significantly in both tumours with respect to controls (FPKM values from 18,4 to 646,6 log2FC = 5.1 in sample 554; and FPKM values from 17,8 to 50,2 log2FC = 1.4 in tumour 840). Since *BMI1* is a proto-oncogene capable of inactivating the tumour suppressor Cyclin Dependent Kinase Inhibitor 2 A *(CDKN2A*)^15^, it would be reasonably to think that this fusion should contribute to the low levels of *CDKN2A* that characterize this type of neoplasms.

## Discussion

Recurrent gene fusions, generated by chromosomal rearrangements, are usually considered as hallmarks of cancer and, as such, driver mutations. Therefore, the search for new fusions is a task of maximum interest in oncology. The limitations of cytogenetic analysis to identify these fusions have been clearly solved by the use of Next Generation Sequencing (NGS) and, in particular, by the use of transcriptome sequencing through RNA-Seq with the aid of new fusion-transcript detection-algorithms^10,14,16^.

As indicated, two comprehensive analysis of transcriptome variation, with particular reference to the identification of Single nucleotide variants (SNVs) and gene fusions, has been investigated in T-ALL by exploiting the possibilities of RNA-Seq^7,8^. In these works, the authors detected gene fusions that can explain the overexpression of driver genes such as T-Cell Leukaemia Homeobox 1 (*TLX1*), PLAG1 Zinc Finger (*PLAG1*), *LMO1*, or NK2 Homeobox 1 (*NKX2-1*) and others resulting in novel fusion transcripts. However, the T-LBL has not been extensively analysed from this perspective.

In the present paper we addressed the identification of multiple fusion transcripts in a sample series of T-LBL using three different methods of detection (*TopHat-Fusion*, *Chimerascan* and *EricScript*) whose performance has been recently established^14^ (Supplementary Table S2). The total number of fusions could be reduced to only 55 if we only consider those identified by at least two detection methods (Table 1). Among them, only four were fusion transcripts previously described in T-cell lymphoblastic leukaemia/lymphoma (*RIC3-TRBC2*, *PICALM-MLLT10*, *MLLT10-PICALM*, *and ZMYM2-FGFR1*), but most of them are absolutely novel not only in T-cell lymphoblastic neoplasms but also in any type of cancer. Interestingly, 17 fusions had been detected exclusively in non-neoplastic or normal tissues. The fact that 13 out of the 55 fusion transcripts have already been described in some type of cancer reinforces their importance as tumour-causing drivers or at least as tumour-maintaining passengers^17^. Of note, most lymphomas exhibited two or more fusion transcripts. Since many fusion transcripts result from the transcription of fusion genes generated at DNA level, the number of significant fusion transcripts in tumours suggests very sharp differences between the levels of genomic instability between tumours. On the other hand in the two control samples the number of fusion transcripts indicates that the level of genomic instability in controls may be even higher than expected (Fig. 1). Notably, we confirmed the existence of 24 predicted novel fusions that had not been described in cancer or normal tissues yet, indicating a good accuracy of the prediction. Some of the confirmed fusions could explain the overexpression of driver genes such as *COMMD3-BMI1*, *LMO1*, *TAL1* or *JAK3* (Fig. 2).

Five fusions found exclusively in tumour samples could be considered pathogenic (*NFYG-TAL1*, *RIC3-TCRBC2*, *SLC35A3-HIAT1*, *PICALM MLLT10 and MLLT10-PICALM*). However, other fusions detected simultaneously in normal and tumour samples (*JAK3-INSL3*, *KANSL1-ARL17A/B and TFG-ADGRG7*) could be germ-line fusions genes involved in cancer predisposition.

The *NFYC-TAL1* fusion is an intra-chromosomal fusion transcripts that juxtaposes the 5′-UTR region of nuclear transcription factor Y, gamma (NFYC) to the exon 3 of the T-cell acute lymphocytic leukemia 1 (*TAL1*), producing a previously unreported in-frame chimeric transcript. *TAL1* is a transcription factor that can function as an oncogene in T-ALL, which is usually activated by chromosomal rearrangements that place this gene under the control of potent regulatory sequences^6^. Since we have detected a significant increase in the expression levels of *TAL1* and *TP53* in the tumor 521 and *NFYC* is a direct target of TP53 (https://pathcards.genecards.org/card/direct_p53_effectors), it could be speculated that the chromosomal rearrangements that originates this fusion should operate at the genomic level in such a way that overexpression of *TP53* could be responsible for the overexpression of the chimeric transcript.

Another interesting point is that some fusions (as is the case of *KANSL1-ARL17A/A2*, *TFG*-*ADGRG7* and *JAK3-INSL3*) had been also reported in normal tissues. Although this fact could raise some concerns for their potential as biomarkers, the importance of recurrent fusions in non-neoplasic human tissues should not be dismissed or underestimated. To the contrary, they may also result in dramatic reduction in normal cell growth and/or motility^11^ so it could be at least tumour-maintaining passengers^17^.

Regarding the *TFG-ADGRGT* fusion transcript, it has been documented that *TFG* gene participates in several oncogenic rearrangements and may play a role in the NF-kappa pathway^18^. Although the presence of this fusion in healthy individuals might indicate that it could be a polymorphic gene fusion^19^, *TFG*- *ADGRG7* is a recurrent fusion that has been also identified in several types of tumours, including myeloproliferative neoplasms^9,20–22^. Furthermore, *TFG* gene has also been reported as a putative metastatic melanoma tumour suppressor^23^. Therefore it cannot be ruled out that the *TFG-ADGRG7* could be at least a tumour-maintaining passenger gene^17^.

The fusions between *KANSL1 and ARL17A/B* had been also identified both in tumours and in normal thymuses^11^. A similar fusion transcript *KANSL1-ARL17A* (isoform 2) had been reported in primary pancreatic tumours and in cell lines derived from different tumours^12,13,24,25^. Notably, this fusion is more frequent in North American patients being rarely detectable in tumours of individuals from Asia or Africa. However, working with the Utah Pedigree 1463 revealed that this fusion may be a familiarly-inherited fusion gene (germ-line fusion-gene), therefore this fusion has been considered as the first cancer-predisposition fusion-gene^13^. Concerning of the possible role of this fusion transcript in tumorigenesis, *KANSL1* gene encodes a nuclear protein that is a subunit of two protein complexes (the MLL complex and NSL1 complex) engaged in histone acetylation (H4K5 specific and H4-K16 specific) and in p53Lys120 acetylation. Therefore, this gene is involved in regulation of transcription, post-translational regulation, and even in chromosome segregation^26,27^. The partner *ARL17A* gene encodes a tumour suppressor gene protein of the ARF family that seems to be relevant in human carcinogenesis^28^. Therefore, it could be speculated that the lack of some functional domains of *KANSL1* may result in reduced activities in histone acetylation, and even in the control of chromosome segregation. But we could also speculate with the consequences of losing the suppressive character of *ARL17A*. Other fusion transcripts involving *KANSL1* have two different partners: *LRRC37A/A2*. The occurrence of these fusions is not surprising since *LRRC37A* partially overlaps with *ARL17B*, and *LRRC37A2* partially overlaps with *ARL17A*, although these genes use different DNA coding-strands.

The *JAK3-INSL3* fusion is a read-trough fusion transcript we identified in tumours 238 and 346. The *JAK3* is a member of the mammalian Janus kinase subfamily (*TYK2*, *JAK1*, *JAK2*, and *JAK3*) that is commonly expressed in bone marrow and thymus and is involved in T-ALL^6^. By contrary, *INSL3* encodes a member of the insulin-like hormone superfamily that is mainly produced in gonadal tissues. Thus, a hypothesis emerges that this fusion could be a mechanism for *JAK3* to escape the regulation by microRNAs. Since 3′UTR of *JAK3* is longer than the 3′UTR of *INSL3* (1952 bp vs. 342 bp) and has much more recognition sites for multiple miRNA (31 vs. 4 sites for miRNA broadly conserved among vertebrates) (http://www.targetscan.org/cgibin/targetscan/vert_71/; Supplementary Fig. S2), we reasoned that the level of expression of the fusion transcript should be higher than that of the *JAK3* gene alone. As expected, RNA-Seq expression data in tumour 238 showed a significant increase in the levels of expression of the fusion transcript with respect to *JAK3* alone (Supplementary Table S6). Notably, this fusion has been also identified in non-cancer tissues, including lymph node^11^. Therefore, this fusion might be considered as another cancer-predisposition fusion-gene.

Among the new transcript fusions that not encode chimeric proteins we identified the fusion of *RIC3* to *TCRBC2* gene in tumour 460. Interestingly, *RIC3* encodes a member of the Resistant to Inhibitor of Cholinesterase 3-like family, which has never been linked to cancer, so the effect of the fusion is actually causing the over-expression of an adjacent gene, *LMO1*. Since *LMO1* gene is aberrantly expressed in a significant fraction of acute lymphoblastic T-cell leukaemia (T-ALL) as a result of chromosomal translocations^6,29^, it would be reasonable to think that it is this gene and not *RIC3* that is truly responsible for tumour development. Notably, this fusion had been previously detected in T-ALL where it also seems responsible for the over-expression of *LMO1* gene^7^.

The *ZYMYM2-FGFR1* results in the constitutive activation of tyrosine kinase activity of the chimeric protein^30^. The zinc finger protein encoded by *ZYMYM2* gene behaves as a transcription factor and may be part of a BHC histone decacetylase complex. Translocation of this gene with the *FGFR1* gene has been involved in stem cell leukaemia/lymphoma syndrome (SCLL) and a myeloproliferative disorder with use to progress to acute myelogenous leukaemia^31^. Notably, some FGFR1 inhibitors shows a promise to treat patients with kinase fusions involving FGFR1^32^.

Finally, the *COMMD3-BMI1-TRBJ2-1* complex fusion juxtaposes TCR regulatory elements to the *COMMD3-BMI1* protein-coding gene. *BMI1* is a proto-oncogene that encodes a zing-finger protein that is the major component of the polycomb group complex 1 (*PRC1*), and as such, it functions as a chromatin-remodelling repressor of multiple genes including the tumour suppressor *p16* (*CDKN2A*)^15^. Aberrant expression of this gene has been associated with numerous cancers, and may be considered as a biomarker of haematological malignancies^33^. In fact, *BMI1* overexpression has been associated with T-cell lymphomagenesis^34^; whole exome sequencing served to identify *BMI1* mutations in early T-cell precursor-ALL^35^ and *CALM-AF10* + T-ALL expression profiles revealed over-expression of this gene^36^. The gene *COMMD3-BMI1* arises from a read-through transcription between the COMM domain-containing protein 3 and polycomb complex protein *BMI-1* genes that shares sequence identity with each individual gene product^37^. Thus, it is reasonable to think that *COMMD3-BMI1* gene should maintain the oncogenic properties of the *BMI1* gene, and that the over-expression of this complex gene induced by the regulatory elements of *TRBJ2-1* in the *COMMD3-BMI1-TRBJ2-1* fusion should be contributing to the development of T-cell lymphoblastic leukaemia.

In summary, although the size of this T-LBL sample series is very limited, these results suggest a singular landscape of fusions transcripts in T-LBL that is quite different from the one previously detected in T-ALL^7,8^. However, further studies are required to establish the recurrence of these fusions in others sample series of T-LBL and to confirm their functional meaning as new cancer-driver genetic rearrangements.

## Methods

### Primary tumours

A discovery cohort consisting of nine human T-LBL samples and two control thymuses of fetal or paediatric origin without pathology, were used for RNA-Seq analysis. Additionally, 10 primary T-LBL samples and six control thymuses (extended cohort) were used to evaluate the presence of the fusion transcripts identified in the discovery cohort. All samples were provided by the following Spanish Biobanks integrated in the Spanish Hospital Biobanks Network (RetBioH; www.redbiobancos.es): Hospital Universitario Ramón y Cajal-IRYCIS (Madrid), Hospital Virgen de la Salud (Toledo), Complejo Hospitalario de Vigo, Hospital General Universitario Gregorio Marañón (Madrid), Hospital Universitario de La Paz, IdiPaz (Madrid), Hospital Virgen del Rocio (Sevilla), Hospital Infantil San Joan de Deu, SJdD (Barcelona), and IIS-Fundación Jiménez Díaz, (Madrid). Samples and associated data were processed and released following standard operation procedures with appropriate approval by Ethical and Scientific Committees. Lymphomas were diagnosed according to World Health Organization Classification of Hematological Malignancies^38^ and recommendations from the European childhood lymphoma pathology panel^39^. When adequate material was available, additional markers were added to improve the sub-classification of T-LBLs (Supplementary Table S1). Institutional review board approval was obtained for these studies (references CEI 31–773 and CEI-70-1260). The participants provided written informed consent in accordance with the Declaration of Helsinki.

### RNA-seq

Total RNA was obtained using TriPure Reagent (Roche Applied Science, Indianapolis, IN, USA), following manufacturer’s instructions. For massive sequencing of mRNAs RNA integrity Numbers (RIN) were in the range of 7.2–9.8. Image analysis, per-cycle basecalling and quality score assignment were made using Illumina Real Time Analysis software (Illumina, San Diego, CA). BCL files were converted to FASTQ format using Illumina’s Off-Line Basecaller package. Directional RNA-seq libraries resulting from these analyses were sequenced in paired-end format in two different rounds (Illumina HiSeq2000), leading to 50 bp and 76 bp reads (the latter were trimmed to 50 bp). RNA-seq reads were analysed with the next*presso* pipeline^40^. The quality of sequenced reads was checked with FastQC (http://www.bioinformatics.babraham.ac.uk/projects/fastqc/). Alignments to the human genome (GRCh37/hg19) were made with TopHat-2.0.10^41^ using Bowtie 1.0.0^42^ and Samtools 0.1.19^43^. Only two mismatches and five multihits were allowed. Both transcripts assemblies and the estimation of their abundances were calculated with Cufflinks 2.2.1, using the Ensembl GRCh37.74 annotation for human. For practical reasons we have only considered the transcripts isoforms of the gene *TP53* that encode for proteins according to the information showed in Ensembl^44^. Raw sequencing data and transcripts expression quantification is available as a SuperSeries in GEO (Gene Expression Omnibus) under the following ID GSE109234.

### Fusion Transcript discovery

Three different software packages were used to identify candidate fusion transcripts, *ChimeraScan*^45^, *EricScript*^46^, and *TopHat-Fusion*^47^. They differ in the approach used to identify fusions, and show different sensibility and specificity depending on the RNA-Seq properties. This strategy allowed us to obtain three different lists of fusions, with the main focus on common fusion candidates. We have selected these three tools based on a recent benchmark publication^14^ where *EricScript* was appointed as the best tool in tense of sensitivity and positive prediction values (PPV) after the analysis of different RNA-Seq datasets. In fact, *EricScript* and *TopHat-Fusion* did not find any false positive fusions, reaching therefore a PPV of 100%. *ChimeraScan* was selected because, together with EricScript (best-designated tool), it obtained the best results in a set of real RNA-Seq samples, such as the one analysed in this work, through next generation sequencing techniques. We have used the methods with better results in a more real situation. Tophat-Fusion was also successfully used in three previous publications^48–50^. Bioinformatic tools and arguments used are indicated in Supplementary Information.

*Chimerascan*^45^, this Python program uses Bowtie^42^ to align paired-end reads with a merged genome-transcriptome reference. The trimming of the alignment, the identification of discordant sequences, the suggestion of potential chimeras, the junction alignment and the final chimera identification, are accomplished subsequently. In this work we have used *Chimerascan* version 0.4.5 and EnsEMBL GRCh37.74 as a transcriptome reference.

*EricScript*^46^, is a computational framework for the discovery of gene fusions in paired-end RNA-Seq data developed in R, perl and bash scripts. This software uses the BWA^51^ aligner to perform the mapping on the transcriptome reference and BLAT for the recalibration of the exon junction reference. In this study, we have used *EricScript* 0.5.5b and EnsEMBL GRCh37.73 as a transcriptome reference.

*TopHat-fusion*^47^, this utility is used in combination with *TopHat* ENREF 41^41^ for the complete analysis of fusion candidates. It detects fusions by mapping reads to exons, whereas unmapped reads are fragmented into shorter sequences and mapped on the genome. Subsequently, it identifies chimeras if reads fragments map in a solid way with fusions.

The number of reads sequenced per sample and the overall read mapping rate given by TopHat have been included in Supplementary Table S7.

Previous reports about the selected fusion-transcripts identified in the present work were obtained by consulting the Atlas of Genetics and Cytogenetics in Oncology and Haematology (http://AtlasGeneticsOncology.org).

### RT-PCR, Sanger Sequencing

Reverse-Transcription Polymerase-Chain-Reaction (RT-PCR) and Sanger sequencing were used to validate candidate fusion transcripts. RNA were revese-transcribed using first the High-Capacity RNA-to-cDNA^TM^ Kit (Applied Biosystems, Foster City, CA, USA). Sanger DNA sequencing of PCR-amplified fusion sequences were performed with the specific primers indicated in Supplementary Table S2.

## Supplementary information

Supporting Information
