# Supplementary material for: Detection of novel fusion-transcripts by RNA-Seq in T-cell lymphoblastic lymphoma
Source: Sci Rep. 2019 Mar 26;9:5179. doi: 10.1038/s41598-019-41675-3 (PMC6435891; doi:10.1038/s41598-019-41675-3)

Supporting Information

Detection of novel fusion-transcripts by RNA-Seq in T-cell lymphoblastic lymphoma

Pilar López-Nieva[1,2,3], Pablo Fernandez-Navarro[4,5], Osvaldo Graña-Castro[6], Eduardo Andrés-León[7], Javier Santos[1,2,3], María Villa-Morales[1,2,3], María Ángeles Cobos-Fernandez[1,2,3], Laura Gonzalez-Sánchez[1,2,3], Marcos Malumbres[8], María Salazar-Roa[8], and José Fernández-Piqueras[1,2,3].*

[1] Department of Cellular Biology and Immunology. Severo Ochoa Molecular Biology Center (CBMSO). CSIC-Madrid Autonomous University. Madrid. Spain .[2] Institute of Health Research Jiménez Diaz Foundation. Madrid. Spain; [3] Consortium for Biomedical Research in Rare Diseases (CIBERER), Spain. Carlos III Institute of Health. Madrid. Spain; [4] Cancer and Environmental Epidemiology Unit, National Center for Epidemiology, Carlos III Institute of Health. Madrid. Spain; [5] Consortium for Biomedical Research in Epidemiology and Public Health (CIBERESP), Spain; [6] Bioinformatics Unit, Structural Biology and Biocomputing Programme, SpanishNational Cancer Research Center (CNIO), Spain; [7]Bioinformatics Unit, Instituto de Parasitología y Biomedicina "López-Neyra", Consejo Superior de Investigaciones Científicas (IPBLN-CSIC), PTS Granada, Granada, 18016, Spain; [8] Cell Division and Cancer Group, Molecular Oncology Programme, Spanish National Cancer Research Centre (CNIO), Spain; PLN, PFN, OG and EAL. contributed equally to this work

[*] To whom correspondence should be addressed. Phone: +34-911964653 Fax: +34-911964420; Email: jfpiqueras@cbm.csic.es

Table of Contents:

1. **Bioinformatic tools and arguments used**
2. **Legends of Supplementary Tables and Figures**
3. **Supplementary Table S1**
4. **Supplementary Table S4**
5. **Supplementary Table S5**
6. **Supplementary Table S7**
7. **Supplementary Figure S1 (A and B)**
8. **Supplementary Figure S2**

1.- **Bioinformatic tools and arguments used**

(1) Quality check with FastQC
perl /SOFTWARE/FastQC_v0.10.1/fastqc -o outputDir --noextract <input Fastq>

(2) Trimming of 76bp reads to 50bp with seqtk (Version: 1.0-r45)
/SOFTWARE/seqtk/seqtk-master/seqtk trimfq -b 0 -e 26 <input Fastq>

(3) Alignment of reads with Tophat 2.0.10, using Bowtie 1.0.0 and Samtools 0.1.19
export PATH=$PATH:/SOFTWARE/bowtie-1.0.0/:/SOFTWARE/samtools- 0.1.19/

/SOFTWARE/TopHat/tophat-2.0.10.Linux_x86_64/tophat --bowtie1 -p 4 --read- edit-dist 2 --read-gap-length 2 --GTF Homo_sapiens.GRCh37.74.gtf --no- coverage-search --max-multihits 5 --mate-inner-dist 10 --mate-std-dev 80 -- fusion-search --library-type fr-firststrand --read-mismatches 2 --segment- mismatches 1 --segment-length 20 --splice-mismatches 0 -o outputDir /REFERENCES/Homo_sapiens/UCSC/hg19/Sequence/BowtieIndex/genome <sample_R1.fastq> <sample_R2.fastq>

**The genome fasta file (UCSC.hg19) was downloaded from:
https://ccb.jhu.edu/software/tophat/igenomes.shtml

**The Homo_sapiens.GRCh37.74.gtf annotation file was downloaded from:
ftp://ftp.ensembl.org/pub/release-74/gtf/homo_sapiens/Homo_sapiens.GRCh37.74.gtf.gz

**For those samples where trimming of reads was required to adjust read size, trimmed Fastq files where used as input for Tophat.

(4) Quantification of expression with Cufflinks 2.2.1 (cuffquant and cuffnorm)

export PATH=$PATH:/SOFTWARE/samtools-0.1.19/:/SOFTWARE/cufflinks-2.2.1.Linux_x86_64/

cuffquant -p 4 --library-type fr-firststrand --frag-bias-correct /REFERENCES/Homo_sapiens/UCSC/hg19/Sequence/BowtieIndex/genome.fa --multi-read-correct --max-bundle-frags 500000 --seed 123L -o outDir Homo_sapiens.GRCh37.74.gtf <input.bam>

cuffnorm -p 4 --library-type fr-firststrand --seed 123L --library-norm-method geometric -o outDir --labels <label1,..,labelN> Homo_sapiens.GRCh37.74.gtf <sample1.cxb>.. <sampleN.cxb>

**cxb input files for cuffnorm are previously generated by cuffquant (5) Fusion detection with Tophat-Fusion

(5) Fusion detection with Tophat-Fusion

• Database and anottation files obtained from he authors web page at: https://ccb.jhu.edu/software/tophat/fusion_tutorial.shtml

Run fusion detection:
export PATH=$PATH:/SOFTWARE/TopHat/tophat- 2.0.10.Linux_x86_64/:/SOFTW ARE/bowtie-1.0.0/:/SOFTW ARE/samtools- 0.1.19/:/SOFTW ARE/blast/ncbi-blast-2.2.29+/bin:/SOFTW ARE/blast/blast- 2.2.20/bin;

tophat-fusion-post -p 4 --num-fusion-reads 1 --num-fusion-pairs 1 --num-fusion- both 0 --fusion-read-mismatches 2 --fusion-multireads 4 /REFERENCES/Homo_sapiens/UCSC/hg19/Sequence/BowtieIndex/genome

(6) Fusion detection with EricSript

• Database from GRCh37/hg19 obtained from EricSript download page, at https://sites.google.com/site/bioericscript/download

Run fusion detection:
perl ericscript.pl -p 12 -db EricScript_db/ericscript_db_homosapiens_GRCh37/ - -refid homo_sapiens -name <*label*> -o <*output_folder_name*> <sample_R1.fastq> <sample_R2.fastq>

(7) Fusion detection with ChimeraScan

• Get hg19 chromosomes from UCSC:

  o rsync -avzP rsync://hgdownload.cse.ucsc.edu/goldenPath/hg19/bigZips/chromFa.tar.gz .

  o Uncompress it: tar -zxvf chromFa.tar.gz

  o Concatenate all files in 1: cat chr1.fa chr2.fa .... chr??.fa> hg19.fa

• Obtaining transcriptome annotations

  o Downloading transcriptome annotations from UCSC:

    Visit the UCSC Tables page at http://genome.ucsc.edu/cgi-bin/hgTables?command=start
    Set the clade, genome, and assembly flags to match the genome you downloaded
    In the group list select "Genes and Gene Prediction Tracks" From the track list select "UCSC Genes" (or another list of your choice)

From output format select "selected fields from primary and related tables"
In the file type returned box enter a file name of your choice
Click 'get output'. You will be redirected to a second page that allows you to select tables fields to include in the output.

In the upper table (mine reads "Select Fields from hg19.knownGene") select all fields EXCEPT proteinID and alignID.

In the lower table (mine reads "hg19.kgXref fields") select "geneSymbol" and no other fields.

Click 'get output' again. Save the file and note the path.

If you downloaded a compressed (gzipped) transcriptome annotation file, uncompress it as follows:

$ gunzip <genes.txt.gz>

- Running the chimerascan indexer:

python /bin/chimerascan_index.py hg19.fa genes.txt <*index_folder*>

Run fusion detection:
chimerascan_run.py <*index_folder*> <sample_R1.fastq> <sample_R2.fastq> <*output_folder_name*>

2.- Legends of Supplementary Tables and Figures

Supplementary Table S1. - Human primary T-LBLs and control samples provided by Spanish Biobanks.

Supplementary Table S2. - Total significant fusion-transcripts identified by using the three methods of detection: *TopHat-Fusion*, *EricScrip* and *ChimeraScan*.

Supplementary Table S3. - Accurate details of the characteristics of the 55 selected fusions detected by at least two different detection methods in the same sample. In the columns headed by sample codes (C-M), the letter t is the abbreviation of *TopHat-Fusion*, e represent *EricScript*, and c, *ChimeraScan*. The numbers 0 and 1 indicate negative and positive detections. The presence/absence and topography of each fusion in the Atlas of Genetics and Cytogenetics in Oncology and Haematology (URL http://AtlasGeneticsOncology.org) is indicated in the columns V and W.

Supplementary Table S4. - Primers list. All primers used in this study. In those instances in which no reference is indicated, primers were our own design using the Primer3 software (http://frodo.wi.mit.edu/cgi-bin/primer3/).

Supplementary Table S5.- Novel fusions confirmed by Sanger-sequencing. **Positives (grey) and negatives (white), indicating PCR validation of fusion transcripts.** Dark yellow indicate fusions confirmed for all the samples (both tumor and control samples). N.D. Not Done.

Supplementary Table S6.- Detailed description of the fusion transcripts identified by the *EricScrip* algorithm, with indication of break points, DNA strands, fusion-type, junction sequences and gene expression data.

Supplementary Table S7.- Number of reads sequenced per sample and the overall read mapping rate given by TopHat.

Supplementary Figure S1.- Validation of the fusion junction sequences of the novel fusion transcripts by Sanger sequencing. A., Fusions confirmed in all samples. B, fusions confirmed in only a fraction of tumour and control samples. Vertical black-bars indicate the fusion junctions. All validations were performed at the transcript level.

Supplementary Figure S2.- The 3´UTR of *JAK3* and *INSL3* with indication of the recognition sites for multiple miRNA according to the TargetScan database (http://www.targetscan.org/cgibin/targetscan/vert_71/; Vikram Agarwal George W Bell Jin-Wu Nam David P Bartel (2015) Predicting effective microRNA target sites in mammalian mRNAs. eLife 2015;4:e05005 doi: 10.7554/eLife.05005).

Supplementary Table S1.

Cohort	Sample ID	Type	Organ	Sex	Age	% Tumor cells	TdT	Pax5	CD3	CD4	CD8	CD2	CD1a	CD34	CD117	MPO	Characterization
Discovery Cohort	**840**	Tumor	Lymph node	Male	Pediatric	70%	+		ic +	-	-	+/-		+	-	-	ProT-immature T-LBL
	**238**	Tumor	Lymph node	Female	Adult	80%	+	-	+	-	-	+/-	-	-	-	-	PreT / Immature T-LBL
	**521**	Tumor	Lymph node	Male	Pediatric	90%	+	-	+/-	+	+	+	+	-	-	-	Cortical / Common T-LBL
	**408**	Tumor	Lymph node	Female	Adult	80%	+/-	-	+/-	+/-	+/-	+	+	-	-	-	Cortical / Common T-LBL
	**192**	Tumor	Lymph node	Male	Adult	90%	+	-	+	+/-	+	+	+/-	-	+	-	pre-T-Cortical / Common T-LBL
	**346**	Tumor	Mediastinum	Male	Adult	95%	+/-	-	+	+	+	-	-	-	+/-	-	Cortical / Common T-LBL
	**460**	Tumor	Lymph node	Male	Pediatric	70%	+	-	+	-	+			-	-		Medullar / Mature T-LBL
	**104**	Tumor	Lymph node	Male	Pediatric		+		+	+/-	+	+					Cortical / Common T-LBL
	**554**	Tumor	Lymph node	Female	Adult	85%	+	-	+	+	+		+	-	+/-	-	T-LBL
	**404**	Control	Fetal thymus														
	**405**	Control	Fetal thymus														
Extended Cohort	**526**	Tumor	Mediastinum	Male	Pediatric	70%	+/-	-	+/-	-							T-LBL
	**829**	Tumor	Lymph node	Male	Adult	88%	+		+					+		+/-	Immature T-LBL
	**188**	Tumor	Lymph node	Male	Adult												T-LBL
	**135**	Tumor	Lymph node	Male	Adult		+		+	+	+	+	+/-	-	+/-		Medullar / Mature T-LBL
	**154**	Tumor	Lymph node	Female	Pediatric	80%		-	+	-	-		-	-			Immature T-LBL
	**685**	Tumor	Mediastinum	Male	Adult	80%	+	-	+	+	+						Cortical/Medullar Mature T-LBL
	**153**	Tumor	Lymph node	Male	Adult	90%	+	+	-	+	+						T-LBL
	**038**	Tumor	Thymus	Male	Pediatric												T-LBL
	**001**	Tumor	Lymph node	Male	Pediatric		+		+	-	-						PreT/ProT Immature T-LBL
	**639**	Tumor	Thymus	Male	Pediatric	80%	+	-	+	-	+/-		-	-	-	-	Medullar / Mature T-LBL
	**402**	Control	Fetal thymus														
	**403**	Control	Fetal thymus														
	**892**	Control	Pediatric thymus														
	**601**	Control	Pediatric thymus														
	**030717**	Control	Pediatric thymocytes														
	**120717**	Control	Pediatric thymocytes														

ic, intra-cytoplasmic

Supplementary Table S4.

Primer sequences RT-PCR and for Sanger DNA sequencing

Target gene	Primer sequences (5'-3')	Size (bp)
TFG-ADGRG7 [1]	ATGAACGGACAGTTGGATCTAA<br>AAGTAAAACCCATATAGGTACTAT	421
JAK3-INL3	GCTCTTCACCTACTGCGACA<br>AGGTCCCAGCGTGAGATTAC	228
KANSL1-ARL17A[2]	AGTGGCATAGCCAACTTGAG<br>CTTCTGGCACCTTTTGTGTT	399
RIC3-TRBC2[2]	CGTACTCCACAGTGCAGAGA<br>AGAGCCCGTAGAACTGGACT	429
ZMYM2-FGFR1[3]	TCCCTGTGCCTGTGTATATCCC<br>GAGGGTCTTCGGGAAGCTCATA	204
COMMD3-BMI1-TRBJ2	AAAAGCGATCGGTCTTAAAAT<br>GTCCCTGGCCCGAAGAAC	152
CLN6-CALML4	CGGCTGCTTTACTGCCTCTA<br>ACGCCATGACGTAACCTTTC	219
GXYLT2-PPP4R2	GAGGCGTCTACCATGACGAT<br>TAGGGTTGGGAGGACCTCTT	248
XPO7-NPM2	CCATGCACCTGTGTTTTGAG<br>GGCTGCATCTTCTTGTCCTC	242
DNAJC4-VEGFB	CCTTCAGGAAGGTGAAGCAG<br>CATGAGCTCCACAGTCAAGG	217
UTP6-COPRS	AAGGAGCAAGAATCCTGCAA<br>GGGCAGGACTGTCATTAGGA	188
TUT1-EEF1G	CTGGAGCCCAGCATAAATGT<br>AGAACGCTGAACGCAAATCT	216
OPN3-CHML	CACCTCCTCCTGGTCAACAT<br>TTGTCCGCCATTTTAGGAAG	184
KANSL1-LRRC37A	TGACCTGGTGCTTCTGTGTC<br>GACTAGCGTTGTTCCCATGTC	182
SAV1-GYPE	GGAGACTCTGGTTCCCGATA<br>GCCACACCAGTGGTACTTGA	197
GALT-IL11RA	CTGTCCGGAAATTCATGGTT<br>GCAGTCACTCCAGGACAACA	226
DNAAF3-TNNI3	AATCAGCTCTGGGCAACACT<br>CGTTTGGAGGGTCAGTGAG	214
SSSCA1-FAM89B	CCTCCTCCAAGACAAACAGC<br>CTTCCCCAGCTCCTCAGACT	178
KANSL1-ARL17B	TCGAATTCGTCAGCAAACAG<br>CATCATTTGTGCCAGTGACC	226
GPC2-GAL3ST4	ACTGGGACACGACCTGGAC<br>GGAAGGGTGAGGTGACAGAG	211
GAL3ST4-C7orf43	TCCCTAGAGGGGCAAAAGAT<br>ACGGTGAGTGGGAAGATGAC	105
DPP6;ACTR3B	CTGGCAAGATCAACACCTC<br>CAGTGTTGCCTGCGTAGC	250
SNX29;PLA2G10	GTCTTTGAACGGGGAGTTTG<br>GGAGTAGCGCTCTGTCTTGG	214
BPTF-LRRC37A2	CAGTTACTGCACGGAAAGCA<br>CGGGCTTGTAACACCTTCAT	114
SPN-QPRT	CCCTTCCATCCTCCAAGAG<br>ACCAAGGCTGCGTAGTTGAG	216
NFYC-TAL1	GTTCTCCGTGACGCACACT<br>GGGGAAGGTCTCCTCTTCAC	185
PTCRA-CNPY3	CTGAGGGTCACAGCAGGAGT<br>AAAGGCTGACTTCAGCTCCA	125
PPRC1-NOLC1	GCCCTTTGATCTCTGCTTTG<br>GCCTGTAACTTTCGCTCTGG	125
KANSL1-LRRC37A2	TGACCTGGTGCTTCTGTGTC<br>GACTAGCGTTGTTCCCATGTC	182
DTX2-UPK3B	GCCAGTGCTACCTTCCAGAC<br>ATGTAGTGGCCATCGGTCAG	117

Primer sequences for Sanger DNA sequencing

Target Gene	Sequence 5'-3'	Size
KANSL1-ARL17A[2]	TCATCCACAGAGGAGTCACTTAGG<br>AAGTTCAGTTCCCGGCTGG	517

References:

1. Chase A, Ernst T, Fiebig A, Collins A, Grand F, Erben P, Reiter A, Schreiber S, Cross NC. TFG, a target of chromosome translocations in lymphoma and soft tissue tumors, fuses to GPR128 in healthy individuals. Haematologica 2010;95: 20-6.

2. Atak ZK, Gianfelici V, Hulselmans G, De Keersmaecker K, Devasia AG, Geerdens E, Mentens N, Chiaretti S, Durinck K, Uyttebroeck A, Vandenberghe P, Wlodarska I, et al. Comprehensive analysis of transcriptome variation uncovers known and novel driver events in T-cell acute lymphoblastic leukemia. PLoS Genet 2013;9: e1003997.

3. Buijs A, van Wijnen M, van den Blink D, van Gijn M, Klein SK. A ZMYM2-FGFR1 8p11 myeloproliferative neoplasm with a novel nonsense RUNX1 mutation and tumor lysis upon imatinib treatment. Cancer Genet 2013;206: 140-4.

Supplementary Table S5

Fusion	Discovery Cohort										
	Characterization									Control	
	ProT	PreT	Cortical				Medullar	T-LBL		Fetal thymus	
	840	238	521	408	192	346	460	104	554	404	405
CLN6;CALML4	N.D										
GXYLT2;PPP4R2	N.D										
XPO7;NPM2	N.D										
DNAJC4;VEGFB	N.D										
UTP6;COPRS	N.D										
TUT1;EEF1G	N.D										
OPN3;CHML	N.D										
KANSL1;LRRC37A	N.D										
SAV1;GYPE											
GALT;IL11RA	N.D										
DNAAF3;TNNI3											
SSSCA1;FAM89B	N.D										
KANSL1;ARL17B	N.D										
GPC2;GAL3ST4	N.D										
DPP6;ACTR3B											
GAL3ST4;C7orf43	N.D										
SNX29;PLA2G10											
BPTF;LRRC37A2	N.D										
SPN;QPRT											
NFYC;TAL1	N.D										
PTCRA;CNPY3	N:D										
PPRC1;NOLC1	N.D										
KANSL1;LRRC37A2	N.D										
DTX2;UPK3B											

Fusion	Extended Cohort															
	Characterization										Control					
	T-LBL										Fetal thymus		Pediatric thymus		Pediatric thymocytes	
	516	829	188	135	154	685	153	038	001	639	402	403	892	601	030717	120717
CLN6;CALML4																
GXYLT2;PPP4R2																
XPO7;NPM2																
DNAJC4;VEGFB																
UTP6;COPRS																
TUT1;EEF1G																
OPN3;CHML																
KANSL1;LRRC37A																
SAV1;GYPE																
GALT;IL11RA																
DNAAF3;TNNI3																
SSSCA1;FAM89B																
KANSL1;ARL17B																
GPC2;GAL3ST4																
DPP6;ACTR3B																
GAL3ST4;C7orf43																
SNX29;PLA2G10																
BPTF;LRRC37A2																
SPN;QPRT																
NFYC;TAL1																
PTCRA;CNPY3																
PPRC1;NOLC1																
KANSL1;LRRC37A2																
DTX2;UPK3B																

Supplementary Table S7

Sample	Number of total reads sequenced per sample	Overall read mapping rate reported by TopHat
554	118840916	96,40%
840	94003166	94,80%
408	88383136	95,20%
405	66730772	94,90%
404	50250596	94,90%
346	57983356	93,40%
460	66188320	94,80%
238	108761560	95,20%
521	64960498	95,50%
192	42618380	93,80%

Supplementary Figure S1.A-

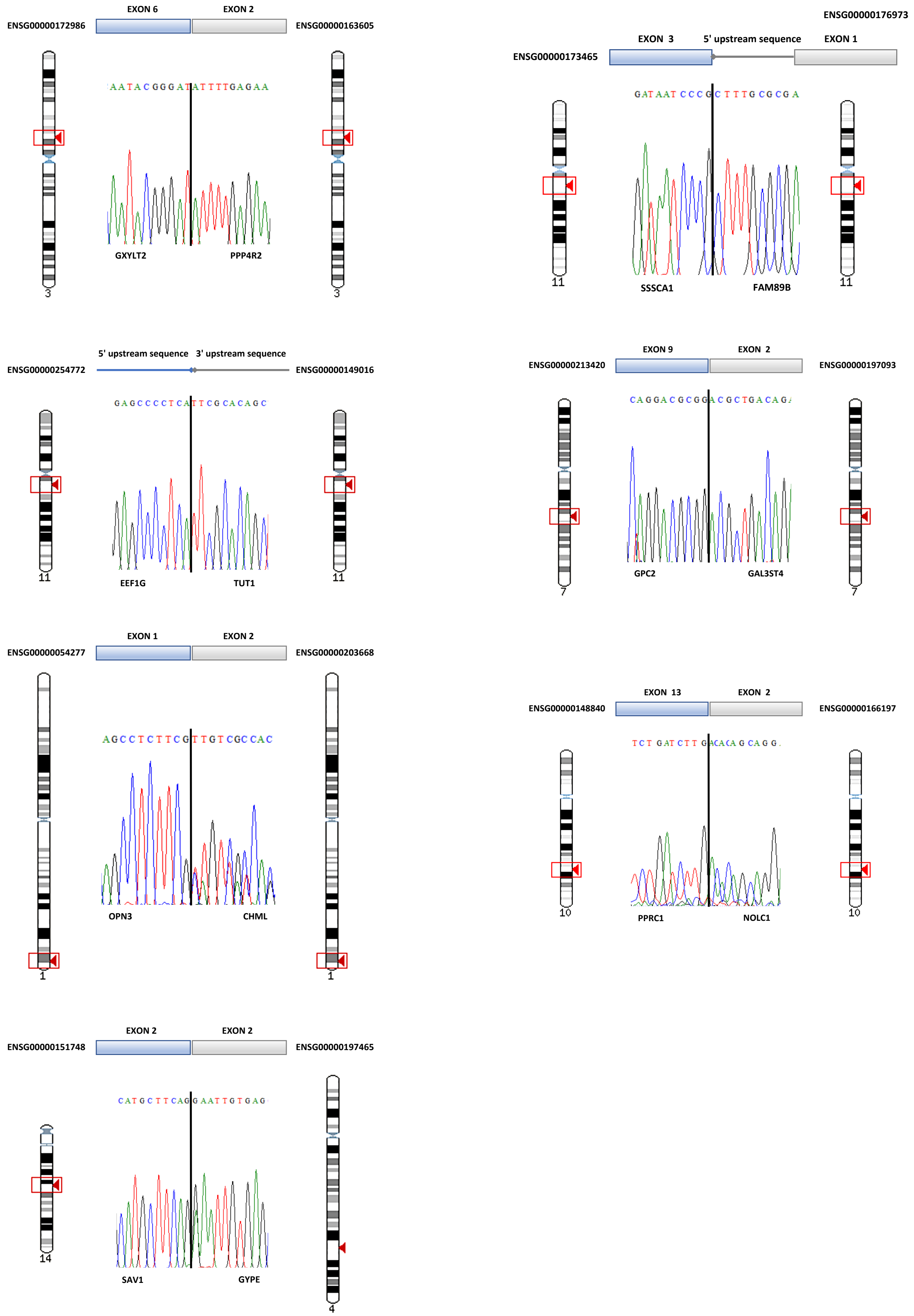

Supplementary Figure S1.B-

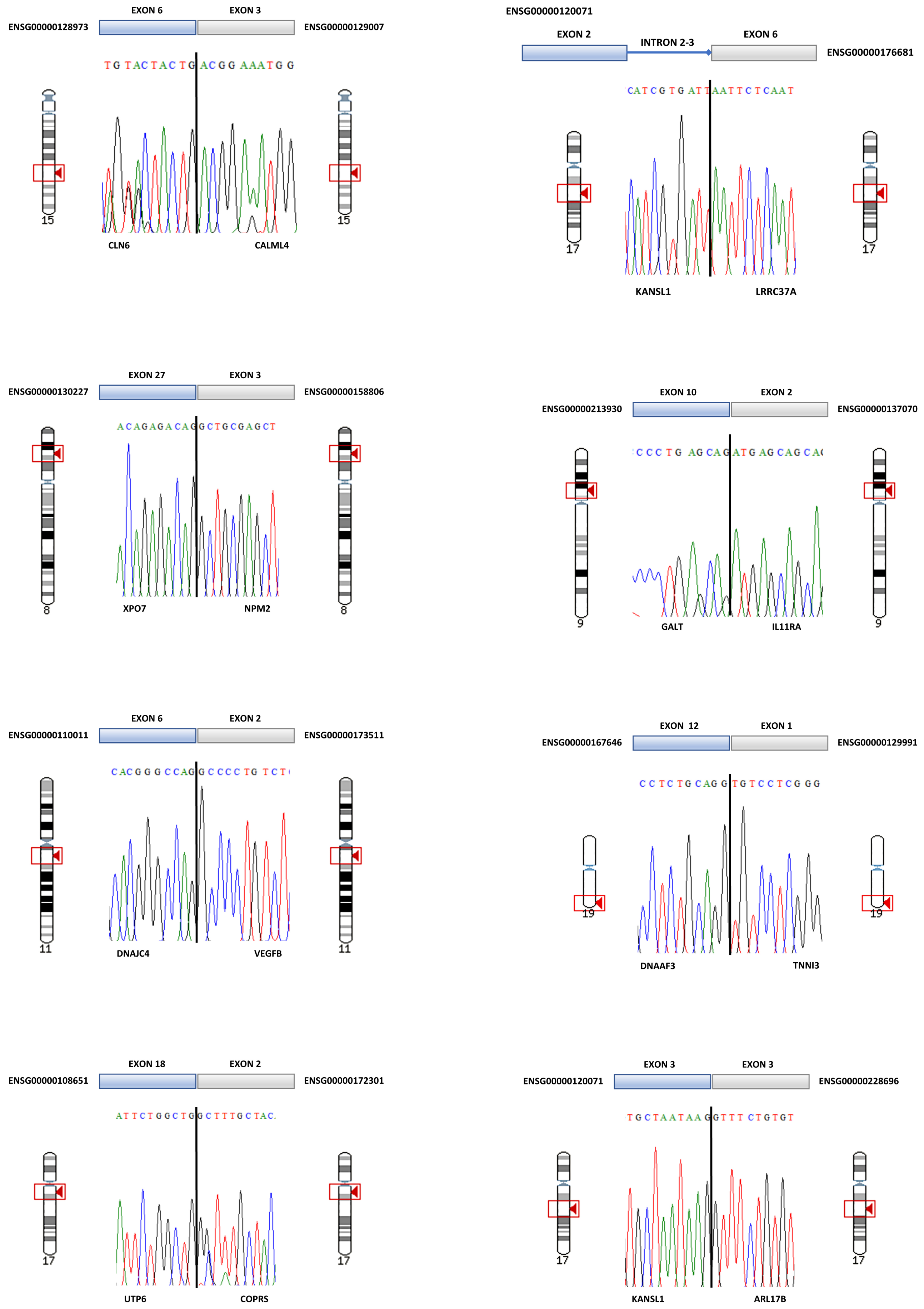

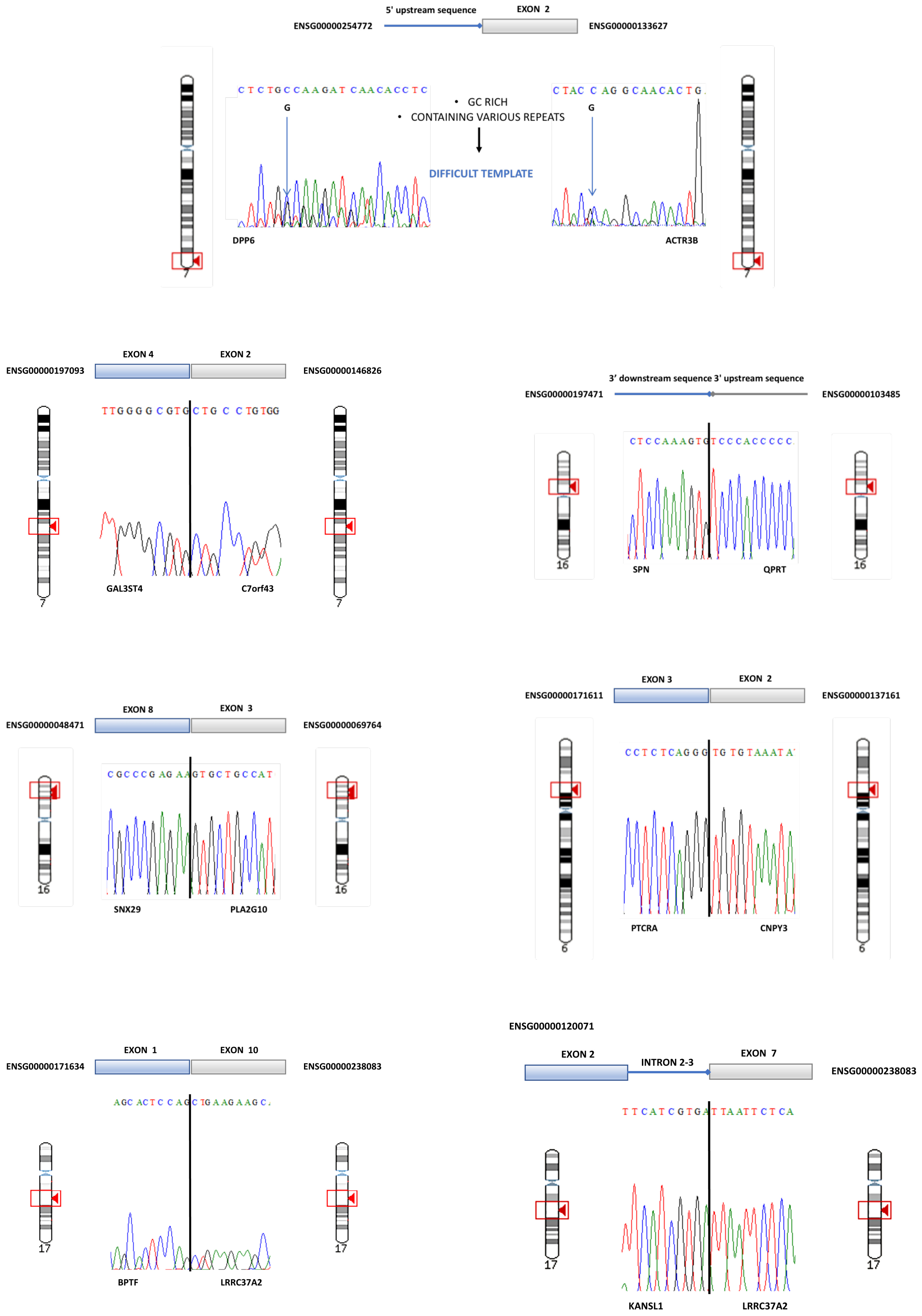

5' upstream sequence
EXON 2
ENSG00000254772
ENSG00000133627
C T C T G C C A A G A T C A A C A C C T C
C TAC C AG G C A A C A C T G
G
G
GC RICH
CONTAINING VARIOUS REPEATS
DIFFICULT TEMPLATE
DPP6
ACTR3B
7
7
EXON 4
EXON 2
ENSG00000197093
ENSG00000146826
T T G G G G C G T G C T G C C T G T G G
GAL3ST4
C7orf43
7
7
3' downstream sequence 3' upstream sequence
ENSG00000197471
ENSG00000103485
C T C C A A A G T G T C C C A C C C C C
SPN
QPRT
16
16
EXON 8
EXON 3
ENSG00000048471
ENSG00000069764
C G C C C G AG A A G T G C T G C C A T
SNX29
PLA2G10
16
16
EXON 3
EXON 2
ENSG00000171611
ENSG00000137161
C C T C T C A G G G T G T G T A A A T A
PTCRA
CNPY3
6
6
EXON 1
EXON 10
ENSG00000171634
ENSG00000238083
AGC ACT C C A G C TG A A G A A G C
BPTF
LRRC37A2
17
17
ENSG00000120071
EXON 2
INTRON 2-3
EXON 7
ENSG00000238083
T T C A T C G T G A T T A A T T C T C A
KANSL1
LRRC37A2
17
17

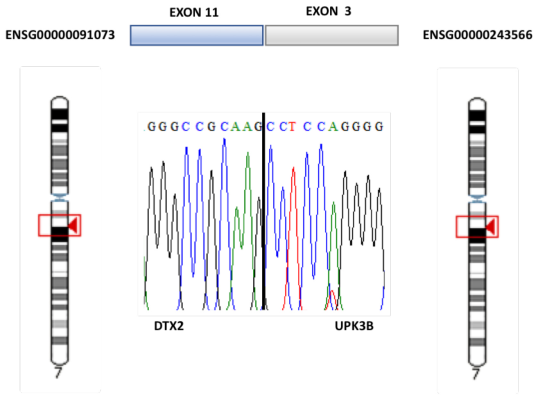
EXON 11
EXON 3
ENSG00000091073
ENSG00000243566
GGGCCGCAAGCCTCCAGGGG
DTX2
UPK3B
7
7

Supplementary Figure S2

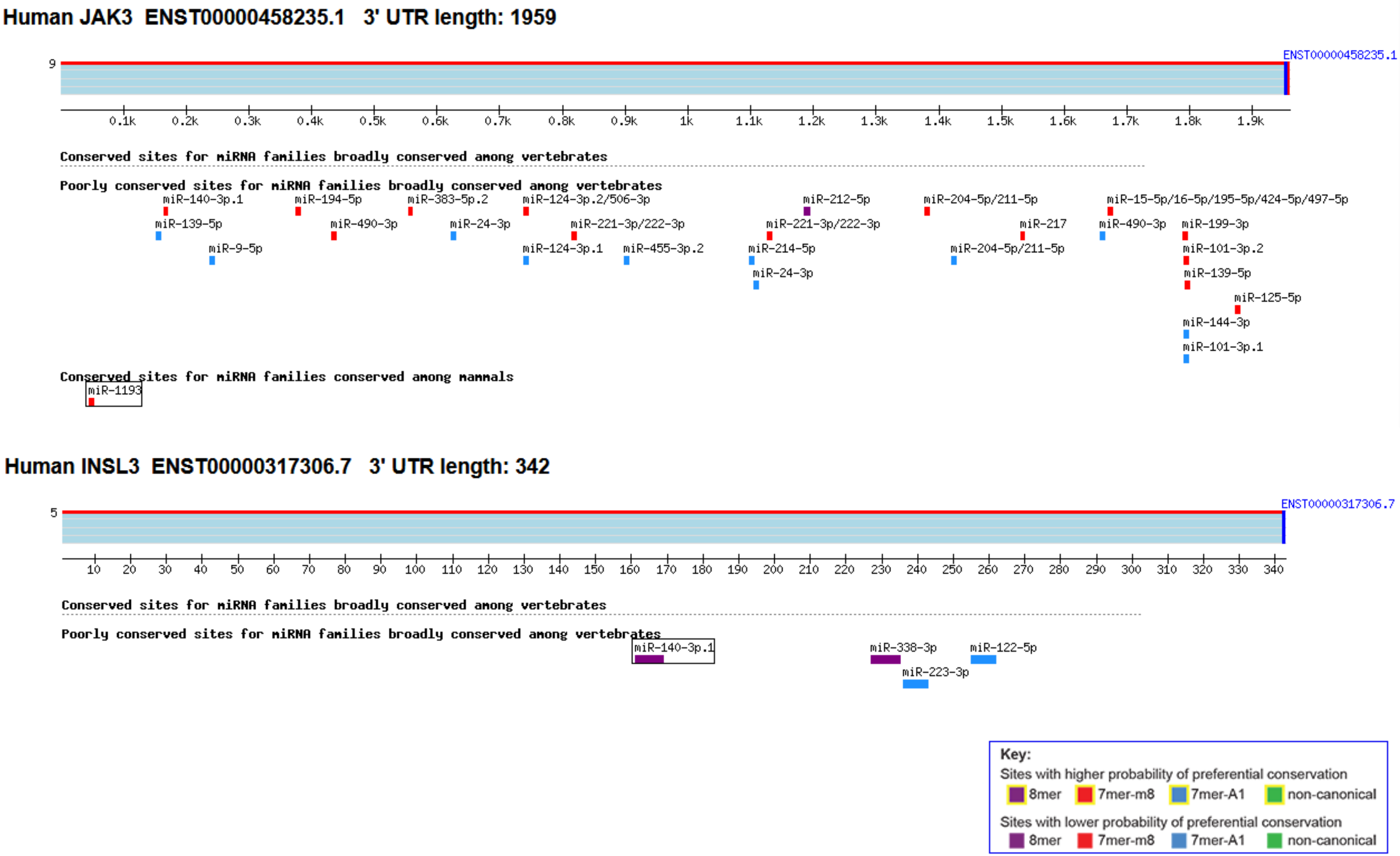

Supplement: Supplementary file 1 — Supporting Information [file 41598_2019_41675_MOESM1_ESM.pdf]
